# Risk scores: A valid tool for reducing mortality in cardiogenic shock?

**DOI:** 10.1002/ehf2.15040

**Published:** 2024-08-29

**Authors:** Gloria Palamara, Alberto Aimo, Daniela Tomasoni

**Affiliations:** ^1^ Cardiology, ASST Spedali Civili and Department of Medical and Surgical Specialties, Radiological Sciences, and Public Health University of Brescia Brescia Italy; ^2^ Interdisciplinary Center for Health Sciences, Scuola Superiore Sant'Anna Pisa Italy; ^3^ Cardiology Division, Fondazione Toscana Gabriele Monasterio Pisa Italy

Cardiogenic shock (CS) is a life‐threatening condition due to primary cardiac dysfunction leading to inadequate cardiac output and peripheral hypoperfusion that finally might result in multiorgan failure.^1^, ^2^ In‐hospital mortality rate ranges between 30% and 60%, depending on the underlying causes, with nearly half of deaths occurring within the first 24 h of admission.^3^, ^4^, ^5^, ^6^ Acute myocardial infarction is the most common aetiology of CS, with mortality rates that remained unchanged over the past 10 years.^5^ Outcomes of CS due to other less common causes, including fulminant myocarditis, right ventricular failure, Takotsubo syndrome, post‐partum cardiomyopathy and end‐stage valvular heart disease, are less defined but generally poor.^7^, ^8^, ^9^

The severity of the disease at initial diagnosis is significantly associated with outcomes. Early risk assessment is therefore crucial for the prognostic stratification and prompt management, including effective monitoring of invasive haemodynamics, timely initiation and appropriate selection of pharmacologic treatments and mechanical support therapies.^10^ The Society for Cardiovascular Angiography and Interventions (SCAI) classification has recently defined 5 evolutive stages of CS, from A (preshock) to E (extremis, i.e., refractory CS).^11^ SCAI shock stages classification provides a robust mortality risk stratification applicable for both acute myocardial infarction related CS (AMI‐CS) and non‐AMI‐CS and has been externally validated in several cohorts.^12^ Additionally, in recent years, several risk scores have been proposed attempting to better describe the different levels of CS severity and aiming to guide clinical decision making.^12^, ^13^, ^14^, ^15^, ^16^, ^17^, ^18^ These scores incorporate a wide range of clinical, laboratory, haemodynamic parameters and demographic variables (*Table* *1*). Most of the selected variables directly correlate with cardiac function and organ perfusion. CardShock predicts in‐hospital mortality across a wide spectrum of aetiologies (although >80% of included patients had AMI),^13^ IABP‐SHOCK II predicts short‐term mortality in AMI‐CS,^14^ SAVE‐ECMO^16^ and ENCOURAGE^15^ assess in‐hospital and 6 months survival in CS patients supported with extracorporeal membrane oxygenation (ECMO). The Cardiogenic Shock Score (CCS) has been recently developed and validated to assess 30‐day mortality risk in CS patients, regardless of the underlying cause, showing the highest C‐index as compared to other scores.^17^ The BOS,MA2, a simple clinically interpretable risk score developed using a novel machine learning approach and validated in external cohorts, predicted in‐hospital mortality with an area under the curve (AUC) of 0.83 (0.82–0.84) and outperformed other existing models.^18^

**Table 1:** Summary of risk scores used for risk stratification in cardiogenic shock

Risk scores	Enrolled population	Main outcome measure	Variables
CardShock^13^	CS (81% with AMI‐CS)	In‐hospital mortality (AUC 0.85)	Age, confusion at presentation, previous MI or CABG, ACS aetiology, LVEF, blood lactate, eGFR (CKD‐EPI)
IABP‐SHOCK II^14^	AMI‐CS	30 day mortality	Age, prior stroke, glycaemia, creatinine, lactate, TIMI flow after PCI
ENCOURAGE^15^	AMI‐CS treated with ECMO	6 months survival to ICU discharge after ECMO	Age, female sex, body mass index, Glasgow Coma Score, creatinine, lactate, prothrombin activity
SAVE‐ECMO ^16^	CS treated with ECMO	In‐hospital mortality (AUC 0.68)	Age, weight, acute pre‐ECMO organ failures, chronic renal failure, aetiology of CS, duration of intubation prior to initiation of ECMO, pre‐ECMO cardiac arrest, peak inspiratory pressure, diastolic blood pressure before ECMO, pulse pressure before ECMO, HCO3 before ECMO
Cardiogenic Shock Score^17^	CS	30 day mortality (AUC 0.740)	Age, female sex, absence of AMI or AMI, SBP, heart rate, pH, lactate, glycaemia, cardiopulmonary resuscitation
BOS, MA2^18^	CS (SCAI stage C or greater)	In‐hospital mortality (AUC 0.83)	Age, blood urea nitrogen, oxygen saturation, SBP, mechanical ventilation, anion gap
SCAI classification^11^, ^12^	CS	Short term mortality	Physical examination/bedside findings, biochemical markers (lactate, creatinine, BNP, LFTs, CPR, pH), haemodynamics (blood pressure, heart rate, CI, PCWP, CVP, PA saturation)
Shock index (SI) and its variants^19^, ^20^	Originally proposed for the management of haemorrhagic and septic shock, and then validated in other settings. Colarusso et al. validate the SI and its variants in patients with CS	In‐hospital mortality	Ratio of heart rate (HR, bpm) to systolic blood pressure (SBP, mmHg). Additional indices include the modified shock index (MSI), defined as the ratio of HR to mean arterial pressure, the age‐adjusted indices (ASI and AMSI) and the shock index‐C (SIC), defined as (SI × 100) minus creatinine clearance.

Abbreviations: ACS, acute coronary syndrome; AMI, acute myocardial infarction; AMSI, age‐adjusted modified shock index; ASI, age‐adjusted shock index; AUC, area under the curve; CABG, coronary artery bypass grafting; CI, cardiac index; CKD‐EPI, chronic kidney disease epidemiology collaboration formula; CRP, C‐reactive protein; CS, cardiogenic shock; CSS, Cardiogenic Shock Score; CVP, central venous pressure; ECMO, extracorporeal membrane oxygenation; eGFR, estimated glomerular filtration rate; ENCOURAGE, prEdictioN of Cardiogenic shock OUtcome foR AMI patients salvaGed by VA‐ECMO; HR, heart rate; IABP‐SHOCK II, Intraaortic Balloon Pump in Cardiogenic SHOCK II score; ICU, intensive care unit; LFT, liver function tests; LVEF, left ventricular ejection fraction; MSI, modified shock index; PA, pulmonary artery; PCI, percutaneous coronary intervention; PCWP, pulmonary capillary wedge pressure; SAVE, Survival After Veno‐arterial‐ECMO; SBP, systolic blood pressure; SI, shock index; SIC, shock index‐C.

Some of these scores were developed in specific populations, potentially limiting their generalizability in real‐world settings. Furthermore, some scores included parameters that are not readily available upon hospital admission and there are scenarios where it might still be too late for effective intervention if the patient deteriorates during transport or is initially managed at a facility lacking advanced therapies. Therefore, there is a critical need for a pre‐hospital risk score that utilizes vital signs and basic clinical assessments, easily obtainable by paramedical staff. Such a score could enhance early identification of high‐risk patients and facilitate their transfer to specialized ‘hub’ centres, potentially improving outcomes. This approach might not only reduce treatment delays and optimize resource allocation but also promote cost‐effectiveness and more efficient healthcare resource management.

In this issue of the journal, Colarusso et al. aimed to respond at this unmet need, evaluating the efficacy of the shock index (SI) and its variants in predicting in‐hospital mortality in patients presenting with CS. Shock index, originally proposed for the management of haemorrhagic and septic shock, is calculated as the ratio of heart rate (HR, bpm) to systolic blood pressure (SBP, mmHg).^19^ Values of SI greater than 1.0 indicate that shock is imminent. Due to its simplicity, SI was previously validated as a prognostic factor for various cardiac conditions, including acute coronary syndrome (ACS), acute heart failure, and out‐of‐hospital cardiac arrest (OHCA). Then, additional indices have been developed, including the modified shock index (MSI), defined as the ratio of HR to mean arterial pressure, the age‐adjusted indices (ASI and AMSI) and the shock index‐C (SIC), defined as (SI × 100) minus creatinine clearance. While these indices were designed to improve predictive accuracy in various settings, their applicability in CS remains to be evaluated.

Colarusso et al. retrospectively included 1282 patients presenting with both ischaemic and non‐ischaemic CS (21.8% being AMI‐CS) and collected shock indices at hospital admission.^20^ The prognostic performance of SI and its variants (MSI, ASI, AMSI and SIC), was analysed using the receiver operating characteristic (ROC) curves, where an AUC of 1.0 indicates perfect discrimination and an AUC of 0.5 indicates random chance. ASI, AMSI and SIC had the highest predictive accuracy for in‐hospital mortality, with AUC values of 0.654, 0.667, and 0.659, respectively. The authors also performed subgroup analyses stratified by age groups (<60 or >60 years), sex, ACS and OHCA to evaluate the discriminatory power of SI indices within specific patient population. Subgroup analyses revealed that SIC had good predictive ability in patients with STEMI (AUC: 0.714) and ACS (AUC: 0.696), while AMSI and ASI were notably predictive in the OHCA group (AUC: 0.707 and 0.701, respectively). In conclusion, SI, and namely its variants, are simple tools for early risk assessment and prediction of in‐hospital mortality in CS patients and their tailored use in specific contexts might be more effective.

The authors' efforts should be recognized, as this is the first study to evaluate SI and its variants in a quite large population of patients with CS. Compared to the previously mentioned validated scores, like the SCAI classification or SAVE‐ECMO, these indices are extremely simple, require fewer parameters that are easily obtainable upon patient admission and do not require invasive measurements. Furthermore, unlike the other scores, which all necessitate at least one laboratory parameter, these indices do not depend on any laboratory measurements, except for SIC. This simplicity enhances the reproducibility and adoption of these measures in any critical care setting, enabling their application shortly after presentation in the emergency department or even before. Indeed, as mentioned previously, these tools could be helpful in a pre‐hospital setting, where even non‐medical personnel can apply them, to direct patients to the most appropriate centre that can guarantee optimal medical therapies, including advanced treatments such as mechanical circulatory support, which are not available in every facility.

Some limitations should be acknowledged when interpreting these data. First, the value of the AUC were non‐adjusted and were not very discriminative. Second, while the simplicity of the score due to its few variables makes it easy to use, it also increases the likelihood of errors. Small variations in parameters, not related to the severity of shock, can lead to incorrect conclusions. For instance, factors such as pain, anxiety and fever might have an influence on SBP and HR, thus affecting the SI and its variants. It would also be beneficial to have information about prior medication use, such as antihypertensive agents or beta‐blockers, that may affect these parameters. Third, the SI and its variants at admission might be influenced by pre‐hospital care. In this study, 40% of CS patients were transferred from another hospital, which could introduce a potential bias, since the SI recorded at the admission might not reflect the true initial presentation. However, the prognostic impact of all indices were confirmed regardless of initial presentation.^20^ Moreover, prior use of inotropes and vasopressors was more frequent among non‐survivors compared to survivors. One could object that these drugs might influence the interpretation of the shock index as well as the prognosis.^20^

In summary, SI and its variant might not fully capture the complexity of a clinical situation such as CS. For this reason, as also stated by the authors, these prognostic tools must be integrated with a throughout evaluation of comorbidities, neurological status, clinical, laboratory and other haemodynamic parameters.

Despite these possible limitations, this study aims to contribute to filling the gap in scientific evidence by providing simple prognostic tools based on easily obtainable variables, offering specific indices tailored to different aetiologies. The study does not support the use of these scores alone to guide therapies or identify those patients who need advanced treatments. The SI score could potentially guide appropriate triage of CS patients, but whether a risk‐score‐guided management might finally influence the prognosis of CS patients, which is particularly challenging, remains to be established. Further research is also necessary to externally validate and compare the SI and its variants with established risk scores for CS.

## Conflict of interest statement

The authors have nothing to declare related to the present manuscript.
